# The prevalence and associated factors of underweight and overweight/obesity among adults in Kenya: evidence from a national cross-sectional community survey

**DOI:** 10.11604/pamj.2020.36.338.21215

**Published:** 2020-08-25

**Authors:** Supa Pengpid, Karl Peltzer

**Affiliations:** 1 ASEAN Institute for Health Development, Mahidol University, Salaya, Phutthamonthon, Nakhonpathom, Thailand,; ^2^Department of Research and Innovation, University of Limpopo, Turfloop, South Africa

**Keywords:** Body weight status, health behaviour, health status, adults, Kenya

## Abstract

**Introduction:**

the study aimed to investigate the prevalence and factors associated with underweight and overweight or obesity in an adult population in Kenya.

**Methods:**

data from a cross-sectional nationally representative community-based study in Kenya (N=4283, 18-69 years) conducted in 2015 was utilized. Assessments included anthropometric, interview, blood pressure and biochemistry mesures. Multinomial logistic regression was used to assess the determinants of underweight and overweight or obesity relative to normal weight.

**Results:**

in all, 11.9% of the study sample was underweight (BMI <18.5kg/m^2^), 60.1% had normal weight (BMI 18.5-24.9kg/m^2^), 18.9% overweight (25.0-29.9kg/m^2^) and 9.1% obesity (BMI ≥30.0kg/m^2^). In adjusted multinomial logistic regression, male sex (adjusted relative risk ratio-ARRR: 1.47, confidence interval-CI: 1.01, 2.13), lower education (ARRR: 0.63, CI: 0.46, 0.88), lower wealth status (ARRR: 0.47, CI: 0.29, 0.78), inadequate fruit and vegetable consumption (ARRR: 1.79, CI: 1.19, 2.70), adding daily sugar into beverages (ARRR: 1.49, CI: 1.01, 2.22) and having no hypertension (ARRR: 0.54, CI: 0.40, 0.74) were associated with underweight. Factors associated with overweight or obesity were middle and older age (ARRR: 2.15, CI: 1.46, 3.80), being female (ARRR: 0.30, CI: 0.22, 0.41), higher education (ARRR: 1.61, CI: 1.26, 2.24), greater wealth (ARRR: 2.38, CI: 1.41, 3.50), being a Kikuyu by ethnic group (ARRR: 1.68, CI: 1.19, 2.37), urban residence (ARRR: 1.45, CI: 1.06, 1.99), no current tobacco use (ARRR: 0.39, CI: 0.24, 0.54), low physical activity (ARRR: 1.49, CI: 1.02, 2.18) and having hypertension (ARRR: 1.96, CI: 1.54, 2.50).

**Conclusion:**

more than one in ten were underweight and almost three in ten were overweight or obese among adults in Kenya. Several risk factors, including sociodemographic, lifestyle and health status risk variables, were identified for underweight and overweight or obesity, which can assist in developing intervention strategies targeting both these conditions.

## Introduction

Worldwide, among adults, 8.8% of men and 9.7% of women are underweight (Body Mass Index=BMI 18.5kg/m^2^) and 10.8% of men and 14.9% of women are obese (BMI ≥30 kg/m^2^) [1]. In the 2014 Kenya “Demographic and Health Survey”, 20.5% and 9.1% of women were overweight and had obesity, respectively [2]. Among adults (40-60 years) in urban slums of Nairobi, 19.6% and 5.1% of men and 30.9% and 32.1% were overweight and had obesity, respectively [3]. There is a dearth of recent national information among men and women on the prevalence and associated factors of underweight and overweight and obesity in Kenya. In Botswana 19.5% of men and 10.1% of women were underweight [4], 23% and 15% of women and 13% and 3% of men were overweight and had obesity, respectively [5], 7.5% were underweight and 31% overweight or obesity among women in Ghana [6] and 27.2% had obesity in South Africa [7]. Moreover, among adults, 6.5% were underweight and 21.9% had overweight and/or obesity in Malawi [8], 5.0% were underweight, 31% overweight and 17% had obesity among men and women in five urban areas in Nigeria [9] and 14.1% were underweight, 16.3% overweight and 4.3% had obesity among women in Addis Ababa in Ethiopia [10].

Having underweight in adulthood can have various negative health effects, including death [11], and having obesity has been associated with different non-communicable diseases (NCDs), including type 2 diabetes and cardiovascular disease, increasing mortality [12]. As reviewed in Pengpid *et al*. [13] factors associated with underweight in adulthood may include male sex, younger or older age, lower socioeconomic status, rural residence, health risk behaviours, such as smoking and insufficient food intake and fear of being obese. Sociodemographic factors associated with overweight or obesity include middle aged [2,14], female sex [8,14], higher socio-economic status [2,14,15], urban residence [2,8,14-16] and ethnicity, being Kikuyu [3]. Dietary risk behaviours impacting on obesity include the “consumption of energy-dense foods high in sugars and fat” [17,18] and insufficient fruit and vegetable intake [19]. Engaging in physical activity [20,21], khat [22] and tobacco use [8,17,18] have been found to decrease the likelihood of having overweight and obesity. Obesity is linked with several chronic NCDs such as hypertension and type 2 diabetes [3,23,24]. Kenya has a high proportion of under-nutrition in children (<5 years) (stunting 26% in 2014) [25], which may negatively affect weight status and NCDs in adulthood. The study aimed to investigate the prevalence and factors associated with underweight and overweight or obesity in an adult population in Kenya.

## Methods

**Data, study design and sample:** in Kenya STEPS survey (2015) 4283 adults (18-69 years) were selected using multi-stage cluster sampling; detailed methods are described elsewhere [26]. The response rate ranged from 93% for biochemical masurements, 95% for questionnaire and 99% for physical and anthropometric measurements [26]. Ethics clearance was obtained from the “Kenya Medical Research Institute´s Ethics Review Committee (SSC No. 2607)” and written informed consent was attained from participants.

**Measures:** following WHO STEPS methodology [27] three steps were taken: step 1: questionnaire interview; step 2: anthropometric and BP measurements; and step 3: biochemistry tests. For step 1, handheld devices loaded with eSTEPS software and WHO STEPS questionnaire were used by trained data collectors at respondents´ residences [27]. Dietary behaviour was assessed with the following questions: consumption of processed food high in sugar (biscuits, wafers, cakes, candy, sweets and chocolate). Responses ranged from 1=always (every meal) to 5=never; consumption of soft drinks (like fanta, coca-cola, 7-up, aya, softa, vimto or other sugary drinks), (responses: number of days in a week and number of servings in one day); adding sugar to your beverages? (Responses ranged from 1=always (every drink) to 5=never); how often do you add salt or a salty sauce such as soya sauce to your food? (Responses ranged from 1=always (every meal) to 5=never); what type of oil or fat is most often used for meal preparation in your household? (Responses ranged from 1=liquid vegetable oil to 10=none used); daily intake of fruit and vegetables (FAV) were calculated from the number of servings consumed per day in a typical week. Insufficient FAV intake was defined as “less than five servings a day” [26]. Physical activity was classified as low, moderate and high, following guidelines from the WHO global physical activity questionnaire [28].

Current tobacco use was measured with two questions, “do you currently smoke any tobacco products, such as cigarettes, hand-rolled, cigars, water pipes/shisha or pipes/kiko” and “do you currently use any smokeless tobacco products such as snuff, chewing tobacco, kuber pan” (yes, no) [26]. Khat use was measured with the item, “do you currently chew Khat” (yes, no) [26]. Binge drinking in the past month was defined as having had ≥6 standard alcoholic drinks in a single drinking session [26]. Body Mass Index (BMI) was measured from scientific body height and weight measures. “The height measurements were taken in centimetre by using a portable height measuring equipment using unique ultrasonic and infrared technology. Weight measurement was done in kilogram using a portable electronic weighting scale” [26]. Pregnant women were excluded. Body weight categories were “underweight (<18.5kg/m^2^), normal weight (≥18.5-24.9kg/m^2^), overweight (≥25-29.9kg/m^2^) and obesity (≥30 kg/m^2^)” [29]. Blood pressure (BP) was averaged from three times taken and raised BP was defined as “systolic BP ≥140mmHg and/or diastolic BP ≥90mmHg or where the participant is currently on antihypertensive medication” [30]. Diabetes was defined as “fasting plasma glucose levels ≥7.0mmol/L (126mg/dl); or using insulin or oral hypoglycaemic drugs; or having a history of diagnosis of diabetes” [31].

**Data analysis:** the sample is described with statistics on frequency and weighted prevalence of BMI and other variables. Differences in proportion were tested with Pearson chi-square tests. Multinomial logistic regression was used to assess the determinants of underweight and overweight/obesity (with normal body weight status as reference category). Multi-collinearity was checked; no variable exceeding a value of 1.8. Missing values (<5%) were excluded from the analysis. P <0.05 was considered significant. All statistical procedures were adjusted for complex sample design and conducted with “STATA software version 13.0 (Stata Corporation, College Station, TX, USA)”.

## Results

**Sample and body mass index status characteristics:** the sample comprised 4283 persons 18-69 years (females=58.9%; median age 39 years, IQR=22) from Kenya. In all, 11.9% of the study sample was underweight (BMI <18.5kg/m^2^), 60.1% had normal weight (BMI 18.5-24.9kg/m^2^), 18.9% overweight (25.0-29.9kg/m^2^) and 9.1% obesity (BMI ≥30.0kg/m^2^). The highest proportion of underweight was in the 60 to 69 years age group (19.9% and among men 23.4%) and the 18 to 29 years age group (12.4%), while the highest proportion of obesity was in the 45 to 59 years age group (15.8% and among women 21.2%). Table 1 and Table 2 show sample and BMI status characteristics. In bivariate analyses, BMI status was higher in the older aged, women, urban residence, higher education, greater wealth, being Kikuyu, using liquid vegetable oil for meal preparation, those who were physically inactive, having hypertension and type 2 diabetes. The body weight status was lower in current tobacco and khat users and the body weight status did not differ by dietary behaviour variables (intake of fruit and vegetables, processed food and items containing sugar) (Table 1 and Table 2).

**Table 1:** sample and nutritional status by sociodemographic variables

Variable	Sample	Under-weight	Normal weight	Over-weight	Obesity	Statistic
	N(%)	BMI: <18.5kg/m^2^	BMI: 18.5-24.9 kg/m^2^	BMI: 25.0-29.9 kg/m^2^	BMI: ≥30.0kg/m^2^	P-value
All	4283	11.9	60.1	18.9	9.1	
**Age in years, all**						
18-29	1398(32.6)	12.4	66.0	16.6	5.0	<0.001
30-44	1622(37.9)	9.6	58.1	21.3	11.0	
45-59	855(20.0)	12.6	51.8	19.7	15.8	
60-69	408(9.5)	19.9	48.6	20.8	10.7	
**Sex**						
Female	2523(58.9)	9.5	52.0	24.7	13.8	<0.001
Male	1760(41.1)	14.3	68.0	13.2	4.4	
**Age in years, female**						
18-29	844(33.5)	12.4	58.1	21.9	7.6	<0.001
30-44	920(36.5)	9.3	46.2	28.7	15.8	
45-59	509(20.2)	8.1	46.6	24.2	21.2	
60-69	250(9.9)	12.8	46.0	24.0	17.2	
**Age in years, male**						
18-29	554(31.5)	13.2	75.5	9.6	1.8	<0.001
30-44	702(39.9)	11.8	67.0	16.1	5.1	
45-59	346(19.7)	17.6	55.8	17.3	9.2	
60-69	158(9.0)	23.4	53.2	17.7	5.7	
**Education**						
No/ Primary school incomplete	1752(40.9)	18.6	62.3	12.2	6.9	<0.001
Primary school complete or more	2531(59.1)	8.2	58.9	22.6	10.2	
**Wealth quintile**						
Poorest/Second	1716(40.1)	18.8	65.3	12.6	3.3	<0.001
Middle	863(20.1)	8.9	62.0	19.2	9.8	
Fourth/Richest	1704(39.8)	6.8	54.4	24.7	14.1	
**Ethnic group**						
Other	3638(84.7)	12.7	61.5	17.9	7.9	<0.001
Kikuyu	645(15.3)	7.8	52.8	24.1	15.3	
**Residence**						
Rural	2205(51.5)	14.0	63.5	15.5	7.1	<0.001
Urban	2078(48.5)	8.6	54.8	24.4	12.2	

**Table 2:** sample and nutritional status by health variables

Variable	Sample	Under-weight	Normal weight	Over-weight	Obesity	Statistic
	N(%)	BMI: <18.5kg/m^2^	BMI: 18.5-24.9 kg/m^2^	BMI: 25.0-29.9 kg/m^2^	BMI: ≥30.0kg/m^2^	P-value
**Fruit and vegetable consumption**						
≥5 servings	316(6.1)	7.5	64.9	16.3	11.3	0.164
<5 servings	3952(93.9)	12.2	59.9	19.1	8.9	
**For meal preparation**						
Other	1777(40.2)	13.0	65.0	15.4	6.7	<0.001
Liquid vegetable oil	2472(59.8)	11.4	56.6	21.4	10.7	
**Processed food high in sugar**						
Less than daily or never	4026(93.8)	12.3	60.3	18.7	8.6	0.136
Daily, every meal	254(6.2)	6.9	56.9	21.0	15.2	
**Soft drinks**						
<6-7 days/week	4120(96.0)	12.3	59.8	18.8	9.1	0.336
6-7 days/week	147(4.0)	5.1	64.2	23.3	7.4	
**Add sugar to beverages**						
< Every day/every drink	2676(64.0)	10.6	61.8	17.8	9.8	0.072
every day/every drink	1605(36.0)	14.3	57.2	20.8	7.7	
**Physical activity**						
Moderate/high	3708(89.5)	11.9	61.1	18.8	8.2	0.032
Low	480(10.5)	13.2	51.4	22.2	13.2	
**Current tobacco use**						
No	3748(86.3)	10.3	58.8	20.7	10.2	<0.001
Yes	535(13.7)	22.4	68.5	7.2	2.0	
**Current Khat use**						
No	4001(92.9)	11.7	59.3	19.7	9.3	0.007
Yes	281(7.1)	15.5	71.5	7.5	5.4	
**Past month binge drinking**						
No	3812(86.1)	11.5	59.5	19.4	9.6	0.149
Yes	471(13.9)	14.4	64.1	15.7	5.8	
**Hypertensive**						
No	2877(70.8)	14.0	63.4	16.4	6.2	<0.001
Yes	1401(29.2)	7.0	52.1	24.8	16.1	
**Type 2 diabetes**						
No	3871(97.3)	12.1	60.7	18.4	8.8	<0.001
Yes	142(2.7)	10.1	32.8	32.8	24.8	

**Associations with the prevalence of underweight and overweight/obesity:** factors independently associated with underweight were male sex (Adjusted Relative Risk Ratio-ARRR: 1.47, Confidence Interval-CI: 1.01, 2.13), lower education (ARRR: 0.63, CI: 0.46, 0.88), lower wealth status (ARRR: 0.47, CI: 0.29, 0.78), inadequate fruit and vegetable consumption (ARRR: 1.79, CI: 1.19, 2.70), adding daily sugar into beverages (ARRR: 1.49, CI: 1.01, 2.22) and having no hypertension (ARRR: 0.54, CI: 0.40, 0.74). Factors independently associated with overweight or obesity were middle and older age (ARRR: 2.15, CI: 1.46, 3.80), being female (ARRR: 0.30, CI: 0.22, 0.41), higher education (ARRR: 1.61, CI: 1.26, 2.24), greater wealth (ARRR: 2.38, CI: 1.41, 3.50), being a Kikuyu by ethnic group (ARRR: 1.68, CI: 1.19, 2.37), urban residence (ARRR: 1.45, CI: 1.06, 1.99), no current tobacco use (ARRR: 0.39, CI: 0.24, 0.54), low physical activity (ARRR: 1.49, CI: 1.02, 2.18) and having hypertension (ARRR: 1.96, CI: 1.54, 2.50) (Table 3).

**Table 3:** associations of independent variables with underweight and overweight or obesity (with normal weight as reference category)

Variable	Underweight (<18.5 kg/m^2^)	Overweight/obesity (≥25 kg/m^2^)
	ARRR (95% CI)	ARRR (95% CI)^b^
**Sociodemographics**		
**Age in years**		
18-29	1(Reference)	1(Reference)
30-44	0.76(0.56, 1.04)	2.31(1.58, 3.37)***
45-59	1.01(0.67, 1.52)	2.73(1.98, 3.76)***
60-69	1.57(0.96, 2.59)	2.35(1.46, 3.80)***
**Sex**		
Female	1(Reference)	1(Reference)
Male	1.47(1.01, 2.13)*	0.30(0.22, 0.41)***
**Education**		
No/Primary school incomplete	1(Reference)	1(Reference)
Primary school complete or more	0.63(0.46, 0.88)**	1.61(1.28, 2.24)***
**Wealth quintile**		
Poorest/Second	1(Reference)	1(Reference)
Middle	0.62(0.42, 0.89)*	1.50(1.12, 2.01)**
Fourth/Richest	0.47(0.29, 0.78)**	2.38(1.61, 3.50)***
**Ethnic group**		
Other	1(Reference)	1(Reference)
Kikuyu	0.95(0.56, 1.63)	1.68(1.19, 2.37)**
Urban residence (base=rural residence)	1.30(0.86, 1.97)	1.45(1.06, 1.99)*
**Health variables**		
Fruit and vegetable consumption (<5 servings) (base= ≥5)	1.79(1.19, 2.70)**	1.14(0.87, 1.50)
Liquid vegetable oil for meal preparation (base=other)	1.26(0.99, 1.60)	1.26(0.99, 1.60)
Processed food high in sugar (Daily, every meal) (base= Less than daily or never)	0.80(0.40, 1.63)	1.17(0.68, 2.01)
Soft drinks (6-7 days/week) (base= <6-7 days/week)	0.48(0.15, 1.60)	1.09(0.64, 1.89)
Add sugar to beverages (every day/every drink) (base=< Every day/every drink)	1.49(1.01, 2.22)*	1.26(0.98, 2.18)
Physical activity (low) (base=moderate/high)	1.28(0.74, 2.16)	1.49(1.02, 2.18)*
Current tobacco use (base=no)	1.57(0.98, 2.52)	0.39(0.24, 0.64)***
Current Khat use (base=no)	0.83(0.39, 1.76)	0.62(0.29, 1.33)
Past month binge drinking (base=no)	0.93(0.56, 1.52)	1.03(0.73, 1.45)
Hypertensive (base=no)	0.54(0.40, 0.74)***	1.96(1.54, 2.50)***
Type 2 diabetes (base=no)	1.58(0.64, 1.76)	1.93(0.92, 4.07)

ARRR=Adjusted Relative Risk Ratio; CI=Confidence Interval; ***P<.001; **P<.01; *P<.05

## Discussion

In this nationally representative community-based adult survey in Kenya, the found prevalence of underweight (BMI <18.5kg/m^2^) was 11.9%, overweight (≥25.0 - 29.9kg/m^2^) 18.9% and obesity (BMI ≥30.0kg/m^2^) 9.1%. These findings show a dual burden of underweight (11.9%) and overweight/obesity (≥25kg/m^2^) (28.0%) in Kenya. Similar figures for overweight and obesity were found among women in reproductive age in 2014 in Kenya [2] and for men in urban slums in Nairobi [3]. The prevalence of underweight was in this study among 40-60 year olds urban dwellers (14.8% among men and 7.8% among women) higher than in urban slums in Nairobi (11.7% among men and 3.9% among women), while the prevalence of obesity was higher among female urban slum dwellers in Nairobi (32.1%) [3] than among 40-60 year old female urban dwellers in this study (6.9%) (analysis not shown). The prevalence of underweight in this study in Kenya (14.3% among men and 9.5% among women) compares with the global prevalence among women (9.7%) but is higher in men (8.8%) [1] and compares with prevalence rates in Botswana (19.5% of males and 10.1% of females) [4] and Ghana (7.5% among women) [6] but was higher than in Malawi (6.5%) [8] and in urban areas in Nigeria (5.0%) [9] and was lower than among women in Addis Ababa in Ethiopia (14.1%) [10]. The prevalence of overweight or obesity (≥25kg/m^2^) (4.4% among men and 13.8% among women) in this study in Kenya was for men lower and for women similar to the global prevalence of obesity (10.8% in men and 14.9% in women), but similar to Botswana (3% in men and 15% in women) [5], higher than in Malawi (2.0% in men, 7.4% in women) [8] and lower than in South Africa (27.2%) [7].

The study found that the prevalence of underweight was the highest among 60-69 year-olds (19.9% overall, 23.4% in males and 12.8% in females) and young adults (18-29 years) (12.4%). Previous investigations also found a higher prevalence of underweight among older adults [14,32] and younger adults [32]. In this study, the prevalence of underweight was significantly higher among men than women, which is consistent with some previous studies in Africa [3,4]. In agreement with previous studies [4,14,15,32-34], this study found that the odds for undernutrition decreased with higher education and higher economic background status. Inadequate fruit and vegetable consumption was correlated with underweight, which may be indicative of food insecurity contributing to undernutrition. Consistent with a previous study [35], this study found that having hypertension decreased the odds of having underweight. In terms of overweight or obesity, consistent with previous studies [2,3,8,14-16] this study found that being female, being middle and older aged, having higher educational and economic status, residing in urban areas and being a Kikuyu were associated with having overweight or obesity. One possible reason for this could be that among the different ethnic groups in Kenya the largest epidemiological transition (regarding increase of overweight/obesity and decrease in underweight) has taken place among the Kikuyu in Kenya. Contrary to some previous studies [17-19], this study did not find an association between dietary behaviour and overweight or obesity. Consistent with previous studies [8,17,18,20,21], this study found that physical activity and tobacco use decreased the odds of having overweight and obesity. The possible mechanisms by which cigarette smoking can reduce body weight has been described “by increasing energy expenditure and inhibiting the expected compensatory increase in caloric intake” [36]. The link between obesity and chronic conditions such as hypertension and in bivariate analysis type 2 diabetes was confirmed in this study [3,23,24].

**Study limitations:** the study was limited by its cross-sectional design and apart from blood chemistry and physical measurements all the other information was based on self-reporting, which could have biased responses.

## Conclusion

The study found in a national cross-sectional study that more than one in ten participants were underweight and almost three in ten were overweight or obese among adults (18-69 years) in 2015 in Kenya. Several risk factors, including sociodemographic, lifestyle and health status risk variables, were identified for underweight and overweight or obesity, which can assist in developing intervention strategies targeting both these conditions.

### What is known about this topic

- Overweight or obesity is increasing and underweight decreasing among adults in Africa;
- In the 2014 Kenya “Demographic and Health Survey”, 20.5% and 9.1% of women were overweight and had obesity, respectively.

### What this study adds

- The current study showed that 11.9% were underweight (BMI <18.5kg/m^2^), 18.9% overweight (25.0-29.9kg/m^2^) and 9.1% obesity (BMI ≥30.0kg/m^2^);
- Male sex, lower education, lower wealth status, inadequate fruit and vegetable consumption, adding daily sugar into beverages and having no hypertension were associated with underweight;
- Middle and older age, being female, higher education, greater wealth, being a Kikuyu by ethnic group, urban residence, no current tobacco use, low physical activity and having hypertension were associated with overweight or obesity.
